# *Bacillus subtilis* ANSB168 Producing d-alanyl-d-alanine Carboxypeptidase Could Alleviate the Immune Injury and Inflammation Induced by Ochratoxin A

**DOI:** 10.3390/ijms222112059

**Published:** 2021-11-08

**Authors:** Hanrui Qing, Xueting Huo, Shimeng Huang, Lihong Zhao, Jianyun Zhang, Cheng Ji, Qiugang Ma

**Affiliations:** State Key Laboratory of Animal Nutrition, College of Animal Science and Technology, China Agricultural University, Haidian District, Beijing 100094, China; s20193040584@cau.edu.cn (H.Q.); huoxt@lamic.com.cn (X.H.); shimengh@hotmail.com (S.H.); lihongzhao@126.com (L.Z.); jyzhang@cau.edu.cn (J.Z.); jcanl@cau.edu.cn (C.J.)

**Keywords:** ochratoxin A, mycotoxin, degradation, carboxypeptidase, poultry

## Abstract

Ochratoxin A (OTA) is toxic to animals and threatens food safety through residues in animal tissues. A novel degrading strain *Bacillus subtilis* ANSB168 was isolated and further investigated. We cloned d-alanyl-d-alanine carboxypeptidase DacA and DacB from ANSB168 and over-expressed them in *Escherichia coli Rosetta* (DE3). Then, we characterized the OTA degradation mechanism of DacA and DacB, which was degrading OTA into OTα. A total of 45 laying hens were divided into three equal groups. The control group was fed basal feed, and other groups were administered with OTA (250 μg/kg of feed). A freeze-dried culture powder of ANSB168 (3 × 10^7^ CFU/g, 2 kg/T of feed) was added to one of the OTA-fed groups for 28 days from day one of the experiment. We found that OTA significantly damaged the kidney and liver, inducing inflammation and activating the humoral immune system, causing oxidative stress in the layers. The ANSB168 bioproduct was able to alleviate OTA-induced kidney and liver damage, relieving OTA-induced inflammation and oxidative stress. Overall, DacA and DacB derived from ANSB168 degraded OTA into OTα, while the ANSB168 bioproduct was able to alleviate damages induced by OTA in laying hens.

## 1. Introduction

Ochratoxin A (OTA), produced by several strains of *Aspergillus* and *Penicillium* [1,2,3,4,5,6], is a naturally occurring mycotoxin that commonly contaminates foods and feeds, causing enormous harm to humans and animals. Notably, OTA is defined as a group 2B carcinogen to humans by the International Agency for Research on Cancer (IARC, 1993). According to the European Commission recommendation 2006/576/EC, the maximum tolerable concentration of OTA is 100 μg/kg in poultry’s complete feed. The target organs of OTA are considered to be the kidney and liver. Studies have reported that OTA causes nephrotoxicity, hepatotoxicity, immunotoxicity, teratogenicity, genotoxicity, and carcinogenicity in animals [7,8,9,10]. Plenty of animal feeding trials have been performed to evaluate the potential toxicity of OTA. Injury to kidneys of OTA-treated rats was observed in histological tests, which related to OTA-induced oxidative stress [11]. Immune response and antioxidant defense were affected in the gut and kidney of piglets, owing to long time exposure at a low dose of OTA [12]. Likewise, hepatocyte necrosis was observed in piglets after feeding them OTA-contaminated feed for 33 days [13]. OTA causing oxidative stress and immunosuppression in broiler chicks was also reported, including a significant dose-dependent decrease in superoxide dismutase (SOD), glutathione peroxidase (GSH-Px), total antioxidant status (TAS), immunoglobulin and total antibody titers, phagocytic potential, and lymphoproliferative responses [14,15].

OTA is thermally stable up to 18 °C [16], which means most ochratoxins in agriculture products can pass through industrial processing programs. Consequently, this contributes to the prevalence of mycotoxin contamination, causing enormous economic losses and threatening human health.

Different protocols that are categorized into physical, chemical, and biological strategies have been developed to control levels of OTA in foods and feeds and prevent the toxic effects of OTA in animals and humans [17]. For example, with 3 h of UV irradiation, 81–86% of OTA in feed can be degraded [18], but it requires extra equipment and is time-consuming. Chemical agents, such as ozone, ammonium, bisulfites, and alkaline hydrolysis, are commonly applied in detoxification [15,19]. However, its effectiveness varies and raises problems of chemical residues. Biotechnological strategies are widely used in eliminating OTA because it is environmentally friendly and effective. Several biocontrol strains like *Burkholderia cepacia*, *Brevibacterium casei*, *Aspergillus niger*, and *Alcaligenes faecalis* were researched and evaluated with varying degrees of success [20,21,22,23,24]. The direct degradation of OTA to a less toxic substance ochratoxin α (OTα) is the most significant mechanism [22], but some microorganisms can also have antifungal properties and inhibit OTA production [25,26].

The chemical structure of OTA is 7-carboxyl-5-chloro-8-hydroxyl-3,4-dihydro-3R-methyl-isocoumarin-7-L-β-phenylalanine, which is a phenylalanyl derivative of a dihydroisocoumarin. Between the dihydroisocoumarin moiety and the L-β-phenylalanine, there is an amide bond linkage [27,28]. The enzyme carboxypeptidase can catalyze the hydrolysis of OTA at the amide bond into less toxic OTα and non-toxic L-β-phenylalanine (Phe) [29]. Much of the research in OTA detoxification has examined the effectiveness of a variety of carboxypeptidases. Among them, carboxypeptidases derived from bovine or porcine sources are the most investigated, such as bovine pancreas carboxypeptidase A (CPA, 3.4.17.1) expressed without propeptide and signal peptide [30], bovine pancreas CPA immobilized with a zeolitic imidazolate framework [31], and porcine carboxypeptidase B (CPB, 3.4.17.2) [32]. However, compared with high-cost animal sources, carboxypeptidases from microbes are a more promising prospect. Researchers have studied carboxypeptidases from *Bacillus amyloliquefaciens* ASAG1 [33], *Bacillus subtilis* CW14 [34], and *Lysobacter* sp. CW239 [35]. Although a number of articles have reported different kinds of carboxypeptidases derived from different OTA-degrading strains, the mechanism and purified enzyme of a novel screened strain *Bacillus subtilis* have yet to be researched.

The present study aimed to investigate the detoxification effectiveness and mechanism of *Bacillus subtilis* ANSB168 in vivo and in vitro. The strain ANSB168 in this study was isolated from the cecum of a donkey and could degrade 47.0% of OTA and produce OTα within 18 h in vitro, which led to a hypothesis that ANSB168 has the biological activity of d-alanyl-d-alanine carboxypeptidase. In this study, D-Ala-D-Ala carboxypeptidase DacA and DacB from ANSB168 were cloned and over-expressed in *Escherichia coli Rosetta* (DE3). Moreover, OTA degradation kinetics and the mechanism of recombinant DacA and DacB were characterized. Furthermore, an animal trial was conducted to evaluate the ameliorative effects of the freeze-dried culture of ANSB168 in OTA-fed poultry. The results obtained from this study demonstrate that the strain ANSB168 and its specific enzyme could be applied in OTA degradation.

## 2. Results

### 2.1. Isolation and Identity of OTA-Degrading Bacteria ANSB168

**Figure 1:**
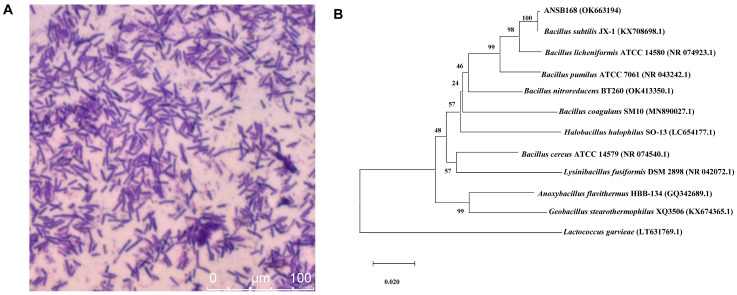
Gram staining and phylogenetic of strain ANSB168. (**A**) Gram staining of ANSB168 (×630). (**B**) Neighbor-joining phylogenetic tree based on 16S rRNA gene sequences, showing the phylogeny position of ANSB168 in genus *Bacillus*. Bootstrap percentages of 1000 replicates are given at branch points, and GenBank accession numbers are given in parentheses. Bar, 1% nucleotide substitutions per 100 nt.

**Figure 2:**
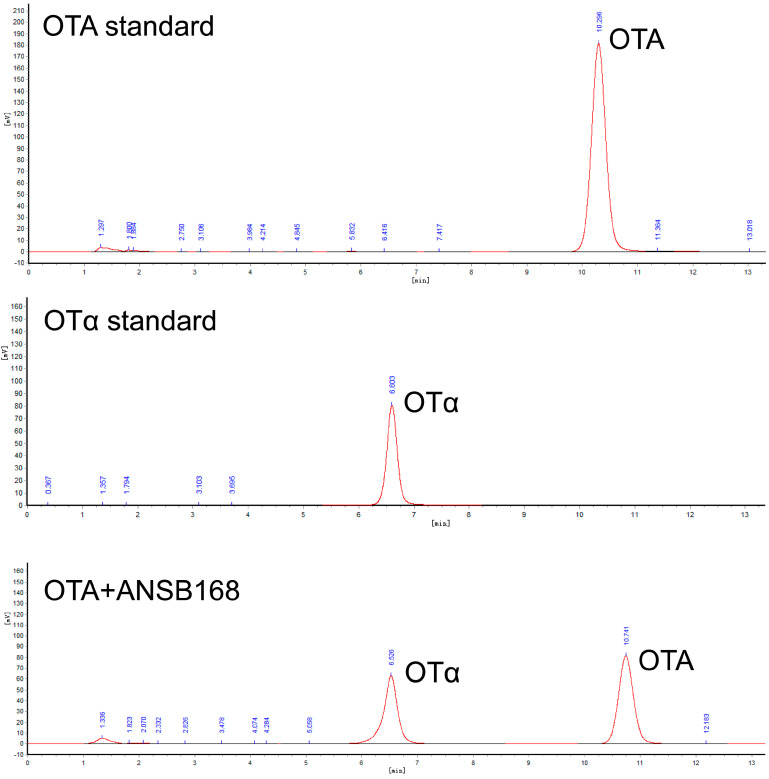
OTA degradation of strain ANSB168, including the High-performance liquid chromatography (HPLC) of OTA standard, OTα standard, and 18 h OTA incubation with ANSB168.

Strain ANSB168 was isolated by the enrichment method from the cecum of donkeys, and the strain showed efficient degradation activity of OTA. The isolated strain ANSB168 is a gram-positive bacillus strain (Figure 1A). The almost complete 16S rRNA gene sequence (1427 bp) of ANSB168 was cloned and analyzed. The constructed phylogenetic tree showed that ANSB168 was a member of the genus *Bacillus* and was in a separate phylogenetic clade with *Bacillus subtilis* (Figure 1B). The 16S rDNA sequence had been uploaded to the National Center for Biotechnology Information (NCBI) GenBank and was obtained with the accession number OK663194. The degradation tests showed that ANSB168 was able to degrade 47.0% of OTA and produce OTα within 18 h (Figure 2), indicating that ANSB168 was capable of breaking the amino bond of OTA. 

### 2.2. Cloning and Expression of DacA and DacB in E. coli

**Figure 3:**
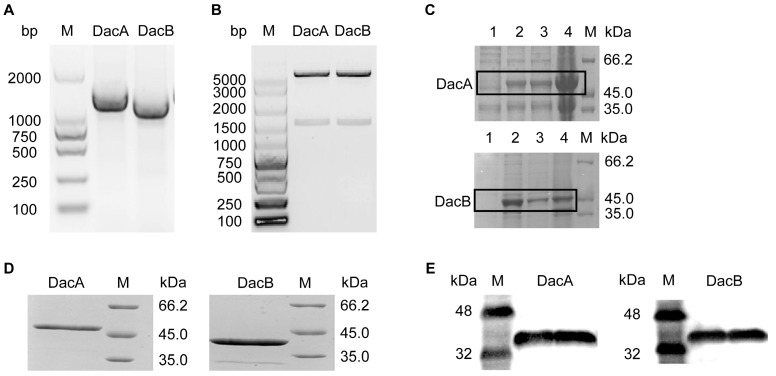
Gene cloning, overexpression, and protein purification of D-Ala-D-Ala carboxypeptidase DacA and DacB. (**A**) Agarose gel electrophoresis of PCR products of gene DacA and DacB. (**B**) Agarose gel electrophoresis of PCR products of gene DacA and DacB from recombinant plasmid. (**C**) SDS-PAGE analysis showing overexpression of the recombinant DacA and DacB in *E. coli* DE3: lane 1, disrupted recombinant cells without IPTG; lane 2, disrupted recombinant cells with IPTG; lane 3, disrupted recombinant cells with IPTG (supernatant); lane 4, disrupted recombinant cells with IPTG (precipitant). (**D**) SDS-PAGE analysis showing the purified DacA and DacB. (**E**) Western blot analysis of recombinant DacA and DacB.

The D-Ala-D-Ala carboxypeptidase DacA and DacB genes were amplified by PCR according to the genomic sequence of ANSB168. sodium dodecyl sulfate-polyacrylamide gel electrophoresis (SDS-PAGE) analysis confirmed the purity of the recombinant DacA with a gene sequence of 1239 bp and DacB with a gene sequence of 1068 bp (Figure 3A). The multiple alignments of the DacA and DacB amino acid sequences were highly homologous to carboxypeptidases. The PCR obtained DNA fragment was purified, digested with NdeI and XhoI, and then ligated into expression vector pET-31b (Figure 3B). The recombinant plasmid pET-31b-DacA/pET-31b-DacB was transformed into *E. coli* Rosseta (DE3), followed by induction with 0.4 mM isopropyl-b-D-thiogalactoside (IPTG) at 37 °C for 3 h. As shown in Figure 3C, *E. coli* Rosseta (DE3)-pET-31b-DacA and *E. coli* Rosseta (DE3)-pET-31b-DacB proteins were efficiently expressed in the supernatant as well as in the precipitant. The expressed recombinant (DacA and DacB) was purified through Ni-NTA column affinity chromatography. SDS-PAGE analysis confirmed the purity of the recombinant DacA with a molecular weight of 46 kDa and DacB with a molecular weight of 41 kDa (Figure 3D). Moreover, purified DacA and DacB were recognized by the mouse anti-His tag antibody by Western blot analysis (Figure 3E). The expression quantities of DacA and DacB were 10.26 mg/L and 9.24 mg/L, respectively.

On the whole, the DacA gene was 1239 bp in length, encoding 412 amino acids, of which its theoretical molecular weight was 45.3 kDa, while the DacB gene was 1068 bp in length, encoding 355 amino acids, and its theoretical molecular weight was 40.1 kDa. The expressed DacA/DacB protein had a histidine tag (6 × His) at the C-terminus. Therefore, the molecular weight displayed on the protein gel chart is slightly larger than that of the prediction.

### 2.3. d-alanyl-d-alanine Carboxypeptidase DacA and DacB Degradation Activity and Biochemical Characteristics

**Figure 4:**
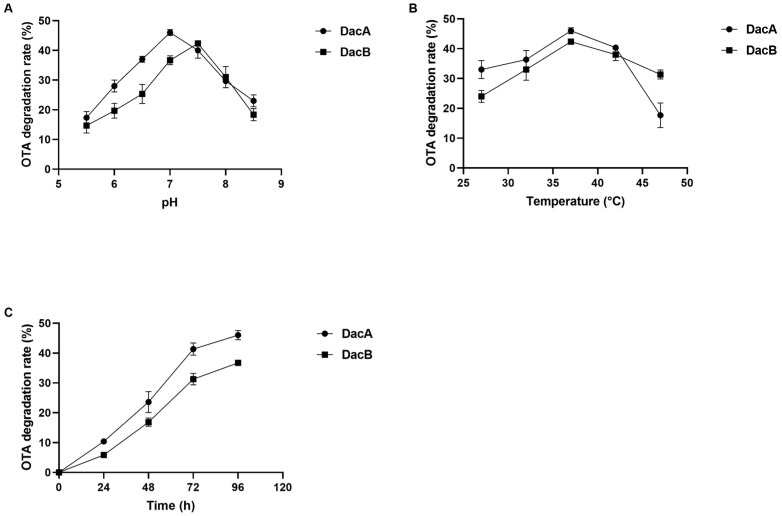
Detoxification characteristics of OTA by DacA and DacB: (**A**) the optimum pH of DacA and DacB; (**B**) the optimum temperature of DacA and DacB; (**C**) the degradation time of OTA by DacA and DacB.

Various buffers at different pH values were used to investigate the optimum pH of the purified recombinant DacA and DacB. The optimum pH values for OTA degradation of DacA and DacB were 7.0 and 7.5, respectively (Figure 4A). Notably, DacA exhibited higher relative degradation activity than DacB when evaluated in pH 5–7, while there were no OTA degradation activities of either enzyme detected at pH below 5.0. The degradation efficiency of DacA was above 35% within 72 h when the pH was between 6.5 and 7.5. When the pH was above 7.5, the activity of DacB to degrade OTA decreased rapidly. 

The optimum pH was used to evaluate the preferred temperature of DacA and DacB. The optimal temperature for OTA degradation was found to be 37 °C for both DacA and DacB (Figure 4B). When the reaction temperature was higher than 42 °C, the OTA degradation activity of DacA decreased significantly. In the range of 32–47 °C, DacB maintained a relatively high OTA degradation activity; the OTA degradation efficiency was higher than 30% after 72 h of incubation. 

The kinetic parameters, Km and Vmax, were 2.74 μg/mL and 73.53 ng/h/mg for DacA and 1.14 μg/mL, and 42.74 ng/h/mg for DacB when determined at 37 °C and optimal pH. The OTA degradation ratio increased over the incubation time. DacA and DacB were able to degrade 45% and 42% of OTA after 72 h, respectively (Figure 4C). 

### 2.4. Degraded Product Identification of DacA and DacB

**Figure 5:**
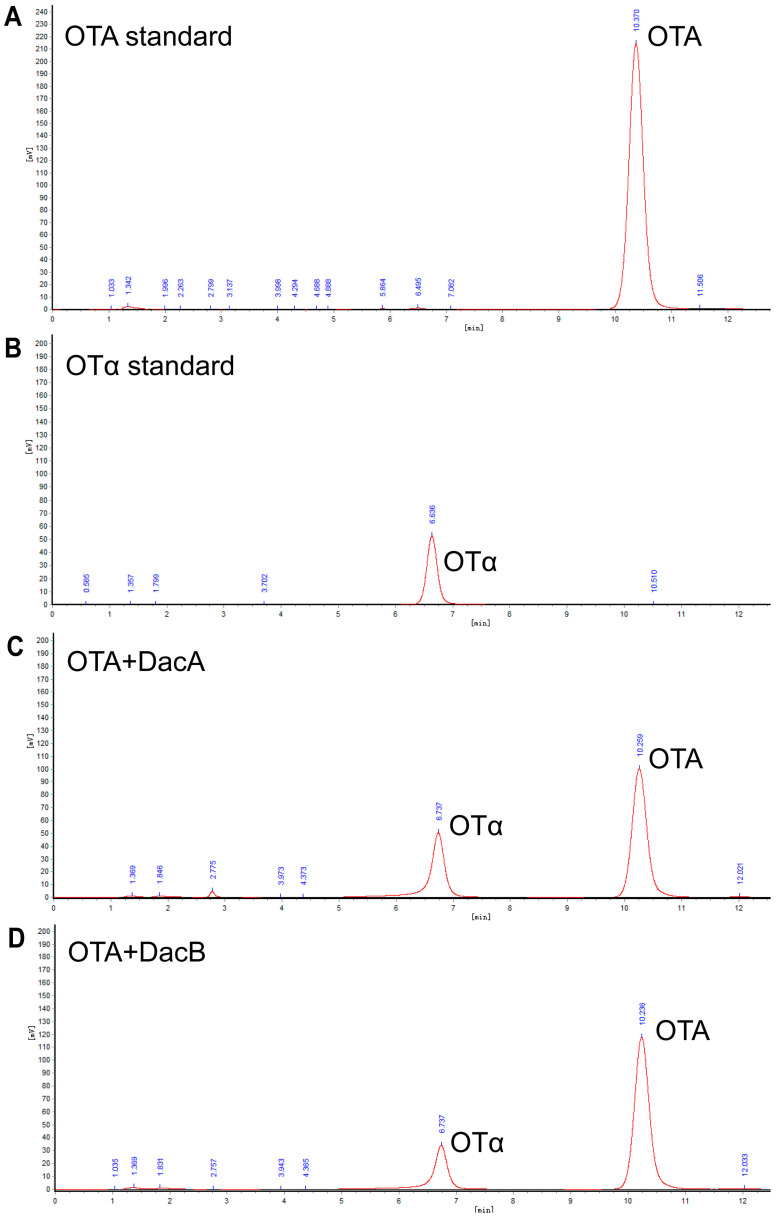
Determination of OTA and OTα by HPLC: (**A**) OTA standard; (**B**) OTα standard; (**C**) OTA standard processed by DacA; and (**D**) OTA standard processed by DacB.

High-performance liquid chromatography (HPLC) analysis indicated that the DacA and DacB degradation products were eluted as a peak with a retention time of 6.7 min that had the same transition time of OTα (Figure 5), suggesting that DacA and DacB break the amide bond of OTA. These results demonstrate that DacA and DacB possess OTA-degrading activity, and the strain ANSB168 can eliminate OTA employing biodegradation.

### 2.5. Ameliorative Effects of Bacillus subtilis ANSB168 in Laying Hens

#### 2.5.1. Production Performance

**Figure 6:**
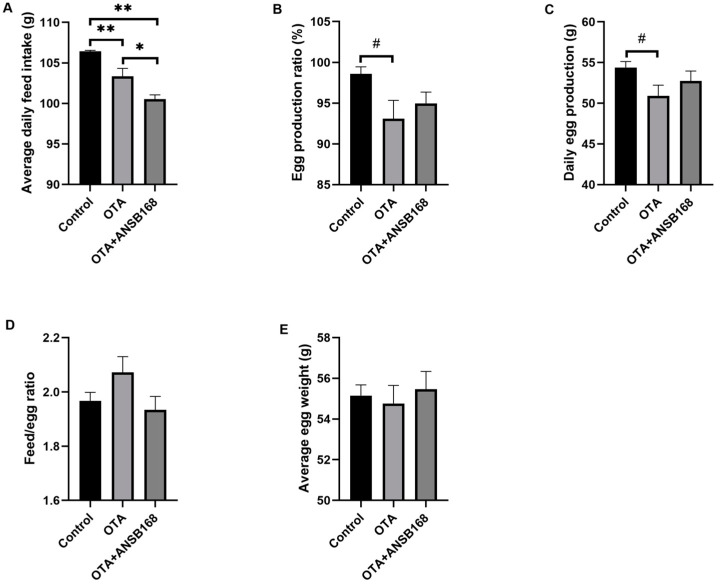
Production performances of layers during the study (*n* = 15, mean ± SEM). (**A**) Effects of OTA and ANSB168 on layers’ average daily feed intake (g). (**B**) Effects of OTA and ANSB168 on layers’ egg production ratio (%). (**C**) Effects of OTA and ANSB168 on layers’ daily egg production (g). (**D**) Effects of OTA and ANSB168 on layers’ feed/egg ratio. (**E**) Effects of OTA and ANSB168 on layers’ average egg weight (g). Data were analyzed with ANOVA and Tukey’s HSD and significant differences were defined as # 0.05 ≤ *p* < 0.10, * *p* < 0.05, and ** *p* < 0.01.

Feeding layers with OTA at the concentration of 250 μg/kg had negative effects on laying hens’ measured performance parameters (i.e., egg production ratio, average egg weight, daily egg production, feed/egg ratio, and average daily feed intake). In Figure 6A, the daily feed intake of the OTA-fed group and the OTA+ANSB168 group was significantly decreased compared with the control group (*p* < 0.01). Both the egg production ratio (Figure 6B, *p* = 0.059) and the daily egg production (Figure 6C, *p* = 0.099) of the OTA-fed group had a downward trend compared with the control. When compared with the control group, the egg production ratio and the daily egg production decreased 5.49% and 6.25%, respectively. Although not statistically significant due to the variable differences within groups, the OTA-fed group’s feed/egg ratio increased by 5.28% (Figure 6D) and average egg weight decreased 0.71% (Figure 6E) compared with the control group. However, the OTA+ANSB168 treatment group showed ameliorative effects on feed consumption, egg production ratio, egg production, feed/egg ratio, and egg weight compared with the OTA-fed group (Figure 6B–E).

#### 2.5.2. Serum Biochemical Parameters

**Figure 7:**
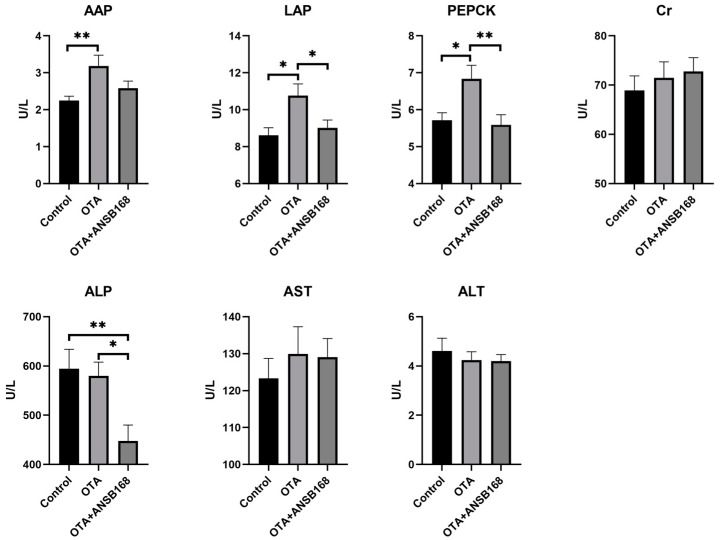
Effects of OTA and ANSB168 on serum biochemical parameters of layers after 28 days (*n* = 15, mean ± SEM), including AAP, LAP, PEPCK, Cr, ALP, AST, and ALT. Data were analyzed with ANOVA and Tukey’s HSD and significant differences were defined as * *p* < 0.05, and ** *p* < 0.01.

The levels of the serum biochemical parameters for the layers kept on different dietary treatments for 28 days have been presented in Figure 7. The levels of the kidney damage parameters alanine aminopeptidase (AAP) (*p* < 0.01), leucine aminopeptidase (LAP) (*p* < 0.05), and phosphoenolpyruvate carboxykinase (PEPCK) (*p* < 0.05) significantly increased in the OTA-fed group compared with the control group. The level of creatinine (Cr) in the OTA-fed group was higher than that of the control group, though it was not statistically significant. As for liver damage parameters, including alkaline phosphatase (ALP), aspartate aminotransferase (AST), and alanine aminotransferase (ALT), there were no statistically significant differences between the OTA and the control group. However, numerically worse ALP and AST could be observed in the OTA group compared with the control. The results elucidate that 250 μg/kg of OTA in feed induced certain kidney and liver damages, and the injury in the kidney was worse than in the liver. 

Moreover, the level of LAP (*p* < 0.05), PEPCK (*p* < 0.01), and ALP (*p* < 0.05) in the OTA+ANSB168 group showed a significant decrease compared with that of the OTA-fed group. Levels of AAP also registered a reduction in the OTA+ANSB168 group, though it was not statistically significant. These results indicate that the ANSB168 bioagent could alleviate the damages to the kidney and liver caused by oral OTA, thereby having benefits for layers.

#### 2.5.3. Oxidative Stress and Antioxidant Status

**Figure 8:**
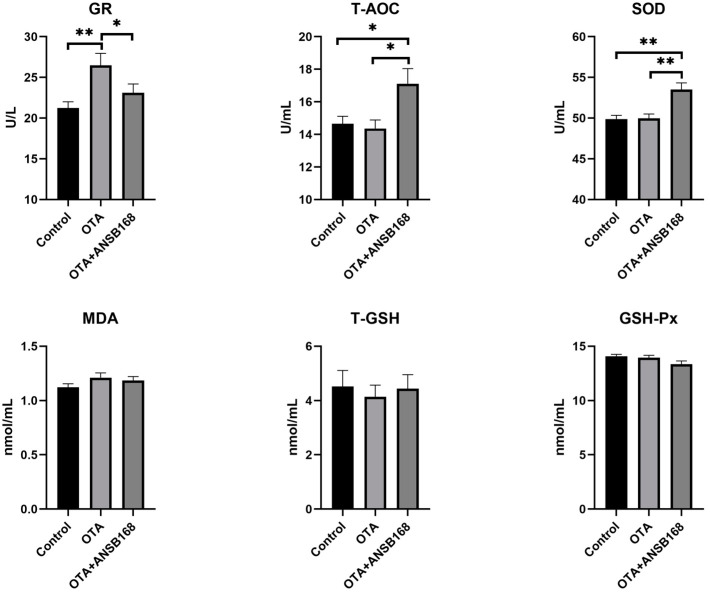
Effects of OTA and ANSB168 on oxidative stress and antioxidant status of layers after 28 days (*n* = 15, mean ± SEM), including GR, T-AOC, SOD, MDA, T-GSH, and GSH-Px. Data were analyzed with ANOVA and Tukey’s HSD and significant differences were defined as * *p* < 0.05, and ** *p* < 0.01.

The oxidative stress and antioxidant status of OTA-fed and ANSB168-fed layers have been presented in Figure 8. The glutathione reductase (GR) level was significantly higher in the OTA-fed group than in the control group (*p* < 0.01), while the OTA+ANSB168 group showed a decrease compared with the OTA-fed group (*p* < 0.05). Plasma total antioxidant capacity (T-AOC) (*p* < 0.05) and SOD (*p* < 0.01) levels were significantly higher in the OTA+ANSB168 group compared with the other groups. The levels of malonaldehyde (MDA), total glutathione (T-GSH), and GSH-Px differed non-significantly among all groups. These results indicate that 250 µg/kg of OTA in the diet could induce layers’ oxidation reaction. However, the layers promoted the regeneration of reduced glutathione by increasing the GR’s activity, thereby alleviating its oxidative stress effects. The supplement of ANSB168 could improve the ability of antioxidants by increasing the activity of T-AOC and SOD, therefore leading to ameliorative effects to OTA-fed layers.

#### 2.5.4. Immune and Inflammatory Response

**Figure 9:**
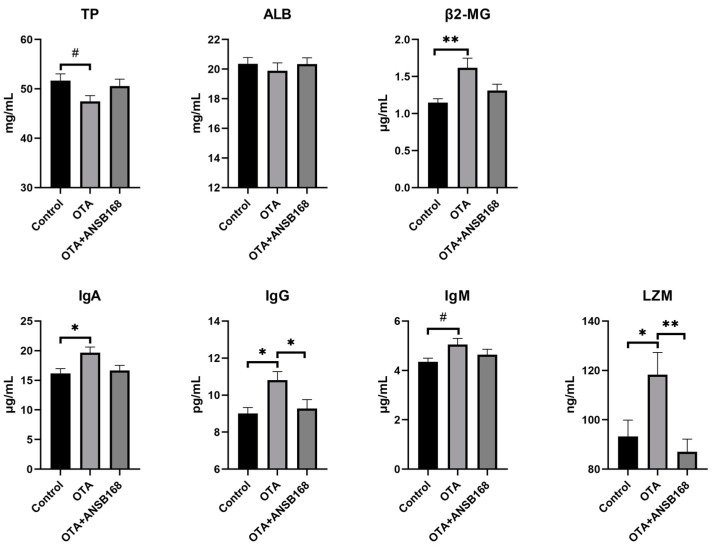
Effects of OTA and ANSB168 on immune response of layers after 28 days (*n* = 15, mean ± SEM), including serum immunoglobulin (TP, ALB, β2-MG, IgA, IgG, IgM) and LZM. Data were analyzed with ANOVA and Tukey’s HSD and significant differences were defined as # 0.05 ≤ *p* < 0.1, * *p* < 0.05, and ** *p* < 0.01.

Figure 9 exhibited the immune response in layers caused by the OTA and ANSB168 diet. Levels of β2-microglobulin (β2-MG) (*p* < 0.01), immunoglobulin A (IgA) (*p* < 0.05), immunoglobulin G (IgG) (*p* < 0.05), and lysozyme (LZM) (*p* < 0.01) in the serum after OTA feeding showed a significant increase compared with the control group. In addition, an increasing trend of immunoglobulin M (IgM) levels (*p* = 0.065) and a decreasing trend of total protein (TP) levels (*p* = 0.068) were observed in OTA-fed layers, compared with the control. Meanwhile, the levels of IgG (*p* < 0.05) and LZM (*p* < 0.01) in the OTA+ANSB168 groups were significantly lower than that of the OTA-fed group. When compared with the OTA-fed group, β2-MG, IgA, and IgM levels in the OTA+ANSB168 groups were lower and the level of TP in the OTA+ANSB168 group was higher. There was no significant difference in albumin (ALB) among all groups.

**Figure 10:**
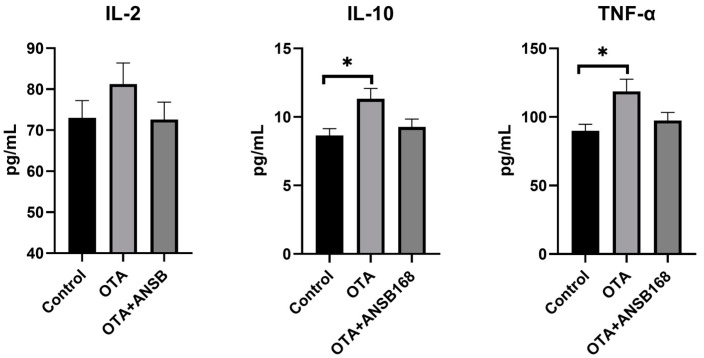
Effects of OTA and ANSB168 on serum cytokines (IL-2, IL-10, and TNF-α) of layers after 28 days (*n* = 15, mean ± SEM). Data were analyzed with ANOVA and Tukey’s HSD and significant differences were defined as * *p* < 0.05.

The inflammatory responses in the layers are shown in Figure 10. In the OTA-fed group, the levels of interleukin-10 (IL-10) and tumor necrosis factors-α (TNF-α) were significantly higher than those of the control (*p* < 0.05). Apart from that, the concentration of interleukin-2 (IL-2) was higher than that of the control, though it was not statistically significant. Overall, they all increased in the OTA-fed group and decreased in the OTA+ANSB168 group. 

These data suggest that the ANSB168 bioagent could alleviate the immune activation effects and inflammatory response caused by oral OTA.

#### 2.5.5. Residues of OTA and OTα in Eggs

We did not detect residues of OTA and OTα above the detection limit (0.1 μg/kg) of the HPLC method in the eggs of all groups.

## 3. Discussion

OTA is one of the most significant mycotoxins that widely contaminate agriculture products. OTA contamination in feed seriously affects the health and production performance of animals, which may also harm people through the food chain. Numerous studies have reported that OTA has renal toxicity, liver toxicity, and carcinogenic and teratogenic effects [7,8,9,10]. In recent years, biodegradation strategies for OTA detoxification have become a promising method. The ability to degrade, absorb, or bind OTA in different microorganisms species, including *Brevibacterium* [21], *Alcaligenes* [22], *Aspergillus* [23], *Saccharomyces* [36], *Cupriavidus* [37], and *Lactobacillus* [38], has been examined extensively. In previous research, some *Brevibacterium* spp. strains could degrade 100% of OTA into the nontoxic substance OTα [21]. The strain ANSB168 that we used in this study was initially isolated from the cecum of a donkey and could degrade 47.0% of OTA to produce OTα within 18 h in vitro. However, the mentioned reports did not investigate the efficacy of the purified enzyme in OTA degradation. In our experiment, its degradation mechanism and the melioration effects in poultry were further analyzed.

The degradation of OTA into non-toxic or low-toxic metabolites by microorganisms and their intracellular or extracellular enzymes is the current research hotspot of OTA detoxification in feeds and foods. It has been reported that *Bacillus amyloliquefaciens* ASAG1 [33], *Acinetobacter* sp. neg1 [39], *Bacillus licheniformis* CM21 [40], and *Alcaligenes faecalis* ASAGF 0D-1 [22] can hydrolyze the amide bond in the OTA molecule to generate OTα and Phe. Therefore, it is conjectured that the same type of protease plays the OTA hydrolysis role in these strains. In a previous study, Chang [33] cloned and expressed D-Ala-D-Ala carboxypeptidase from *B. amyloliquefaciens* ASAG1 and confirmed that D-Ala-D-Ala carboxypeptidase could hydrolyze OTA. In addition, Liuzzi [39] found that adding OTA to the culture medium of *Acinetobacter* sp. neg1 could up-regulate the expression of D-Ala-D-Ala carboxypeptidase PJ15-1540. Furthermore, PJ15-1540 expressed exogenously in *E. coli* had OTA degradation activity. The overall results indicate that the D-Ala-D-Ala carboxypeptidase is the potential molecular basis for the bacteria to hydrolyze OTA. In the current study, based on the genomic sequence of D-Ala-D-Ala carboxypeptidases DacA and DacB according to the NCBI database, the strain ANSB168 maintained by the laboratory was used as a template to successfully amplify DacA and DacB by PCR. The method of the *E. coli* expression system for exogenous expression of DacA and DacB genes was well established. Similarly, Liuzzi [39] successfully cloned the D-Ala-D-Ala carboxypeptidase PJ-1540 derived from *Acinetobacter* sp. neg1 ITEM 17016 and achieved soluble expression in *E. coli*. The amino acid sequence of PJ-1540 shared 29% identity with DacA, while PJ-1540 shared 32% identity with DacB. D-Ala-D-Ala carboxypeptidase derived from *B. amyloliquefaciens* ASAG1 and expressed in *E. coli* had the activity of hydrolyzing OTA and inhibiting the growth of OTA-producing *A. niger* [33]. DacA and DacB, derived from ANSB168 genes, were similar to *B. amyloliquefaciens* ASAG1 carboxypeptidase (amino acid sequence similarity of 81% and 35%, respectively).

Although the recombinant proteins DacA and DacB can be successfully expressed in the *E. coli* expression system, DacA and DacB were partly in the form of inclusion bodies. There are two possible reasons for the formation of inclusion bodies. Firstly, the recombinant proteins DacA and DacB may be expressed too quickly to fold correctly, resulting in the generation of the hydrophobic domain. Secondly, the existence of *E. coli* may have side effects on the proteins DacA and DacB. We only used cell-free soluble recombinant proteins in the supernatant for purification because the inclusion bodies need to be denatured and then renatured in a purification process, which is inefficient and may reduce enzyme activity. The expression levels of DacA and DacB were 10.26 and 9.24 mg/L, respectively, which were higher than the 4 mg/L expressions of bovine pancreatic CPA zymogen in *E. coli* [41]. Since the carboxyl end of the recombinant protein carries a His-tag composed of six histidines, the mouse anti-His monoclonal antibody could be employed to identify whether the purified recombinant protein is the target protein. Western blot analysis found that the purified DacA and DacB can be specifically recognized by the mouse anti-His monoclonal antibody, thus further confirming the successful expression and purification of DacA and DacB in this experiment. 

The results showed that D-Ala-D-Ala carboxypeptidase DacA and DacB derived from ANSB168 had OTA degradation activity. Under the optimum conditions, DacA and DacB can hydrolyze 45% and 42% of OTA, respectively, after 72 h of incubation. This degradation ratio was higher than the 33% degradation of OTA by the Neg1 D-Ala-D-Ala carboxypeptidase PJ15-1540 crude enzyme derived from *Acinetobacter* sp. neg1 [39]. Likewise, the D-Ala-D-Ala carboxypeptidase originated from *B. amyloliquefaciens* ASAG1 could degrade 41% of OTA at 28 °C after 12 h of incubation [33], while the carboxypeptidase cp4 derived from *Lysobacter* sp. CW239 could degrade 36.8% of OTA after 24 h of incubation [35]. The Michaelis constant (*K*_m_) can reflect the affinity of an enzyme for its substrate, while *V*_max_ represents the rate of an enzyme-catalyzed reaction when the substrate concentration reaches saturation [42]. In the present study, the *K*_m_ value of DacA and DacB hydrolyzing OTA were 2.74 μg/mL and 1.14 μg/mL, respectively. The calculated values of the *V*_max_ of DacA and DacB hydrolyzing OTA were 73.53 ng/h/mg and 42.74 ng/h/mg, respectively. In general, the affinity of DacB with OTA was greater than that of DacA, while DacA had a higher reaction velocity than DacB.

Determining the structure and toxicity of the degradation products is the key to evaluating whether the mycotoxin-degrading bacteria or enzymes can be used in actual production. OTα is one of the most ideal degradation products that has been widely reported [21,23,43]. Compared with the control group, the DacA and DacB treatment groups had a degradation product peak with a retention time of about 6.7 min. The retention time of the product is consistent with the retention time of OTα standard. Therefore, it was confirmed that DacA and DacB could hydrolyze the amide bond of OTA to generate low-toxic OTα. Likewise, a series of carboxypeptidases derived from different strains, including *B. amyloliquefaciens* ASAG1 [33], *Lysobacter* sp. CW239 [35], and *Acinetobacter* sp. neg1 [39] could also degrade OTA into OTα. On the whole, the results elucidated the degradation mechanism of the newly isolated strain ANSB168. It is confirmed that the intracellular enzyme D-Ala-D-Ala carboxypeptidase DacA and DacB can hydrolyze OTA on the amide bond into less toxic OTα, which makes it necessary to evaluate the actual use of ANSB168 in an in vivo animal test to develop a novel OTA detoxification biological product in the future.

Stoev [44] reported that the egg production number and egg weight were significantly reduced by 10.77% and 3.49%, respectively, in OTA-fed laying hens with 1 mg/kg feed. Likewise, an OTA-contaminated diet could significantly reduce young chickens’ body weight, average daily gain (g/d), and daily feed intake, even at low levels (100 μg/kg feed) [45]. Similar results were obtained with a 100 μg/kg OTA-contaminated diet in breeder hens, where their body weight, feed intake, and egg production (g) significantly decreased by 1.85%, 0.81%, and 18.41%, respectively [46]. In the animal trial, we intended to investigate the amelioration effects of the freeze-dried bacterial culture powder of ANSB168 (3 × 10^7^ CFU/g, 2 kg/T feed) in OTA-exposure poultry. The dose of OTA fed to the laying hens was 250 μg/kg of feed, which is higher than the recommended upper limit in commercial poultry feed in Europe (100 μg/kg feed, 2006/576/EC). We tested the production performances and serum parameters in laying hens after administering OTA and/or freeze-dried ANSB168 for 28 days. The results showed that OTA had negative effects on laying hens’ production performances, including egg production ratio, daily egg production, feed/egg ratio, and average egg weight, though these were not statistically significant due to the variable differences within groups. Plus, the average daily feed intake was significantly lower than that of the control. These results are consistent with those of other studies. In the present study, the supplementation of ANSB168 represented ameliorative effects on layers’ production performances.

Parameters such as the serum concentrations of several proteins and metabolites and the activity of certain enzymes can be used as sensitive indicators of ochratoxin exposure [47]. Biochemical signs of ochratoxin toxicity reported in the literature in poultry include decreases in cholesterol, TP, ALB, globulin, potassium, and triglyceride levels, and increases in uric acid, creatine levels, activities of serum ALP, and GGT [48]. In the current study, significant increases were observed in serum AAP, LAP, PEPCK, GR, β2-MG, IgA, IgG, LZM, IL-10, and TNF-α concentration in birds exposed to OTA in the diet, indicating that OTA induced kidney damage, oxidative stress, immune response, and inflammation. However, the bioproduct of ANSB168 can significantly reduce the concentration of LAP, PEPCK, GR, IgG, and LZM and increase the concentration of T-AOC and SOD, suggesting a degradation effect of OTA-contaminated feed inside layers’ bodies.

It has been reported that OTA was not detectable in the eggs when laying hens were exposed to a 10 μg/kg and 200 μg/kg OTA diet [49]. After administration of 2 mg/kg of OTA in feed, the residues of OTA in the eggs were still not detectable [48,50]. We did not detect residues of OTA and OTα above the detection limit (0.1 μg/kg) with the HPLC method in the eggs of all groups, which is in line with other studies. However, a conflicting report detected 1 to 8 μg/kg of OTA residues in eggs after administrating a 500 μg/kg and 5 mg/kg OTA diet in laying hens [51]. A great deviation between each analysis was obtained in the conflicting report, suggesting that the OTA passage rate into eggs between different individuals varied significantly.

Except for the crop and gizzard, the pH of poultrys’ digestive tract is above 5.0 [52]. In the present study, the optimal pH for DacA and DacB degrading OTA was between 6.5 and 7.5, which happened to be the range of pH of the small intestine. The passage time for 50% relative cumulation of dry excreta through the poultrys’ total digestive tract was about 32 h [53]. The retention time of dry matter in the crop and gizzard only took up 27–33% of the whole gastrointestinal tract emptying time. Considering that digesta could be held in the small intestine for the longest time [54], we deduced that the degradation of OTA by ANSB168 was mainly performed in the small intestine. The degradation activity of DacA and DacB was inhibited at pH below 5.0 and increased with the increase of pH, which demonstrated that the degradation activity of ANSB168 was inhibited in the crop and gizzard and could be reactivated in the intestinal tract.

## 4. Materials and Methods

### 4.1. Chemicals and Strains

OTA standard and OTα standard were purchased from Sigma (St. Louis, MO, USA) and were prepared in HPLC grade methanol. The strain ANSB168 was initially isolated from donkey cecum and maintained with 20% glycerin at −20 °C in our lab. *E. coli* DH5α and *E. coli* Rosetta (DE3) were purchased from Sangon Biotech (Shanghai, China). The pET-31b vector was obtained from Novagen (Madison, WI, USA). The strain *Aspergillus ochraceus* 3.4412 was purchased from CGMCC (Beijing, China).

### 4.2. Enrichment and Isolation of OTA-Degrading Bacteria from Donkey Cecum

Ten samples were collected from the cecum of different donkeys. About 1 g of each sample was suspended in 9 mL of sterile distilled water and kept at room temperature under continuous shaking at 200 rpm for 6 h. Then, the supernatant was serially diluted in sterile distilled water, and a 150 μL aliquot of each dilution was spread on plates with LB medium and incubated at 37 °C until visible colonies appeared. The single colonies were isolated and subsequently streaked on fresh plates to obtain pure cultures. Then, the cultures were tested for OTA degradation capacity. For bacterial OTA degradation, a final concentration of 2 μg/mL OTA was mixed with a 500 μL LB broth containing 1 × 10^8^ CFU/mL of ANSB168 and incubated for 18 h at 37 °C. After incubation, OTA was analyzed by HPLC. After analysis, the strain ANSB168 showed the highest OTA degradation ability, so it was chosen for further studies.

### 4.3. Cloning of d-alanyl-d-alanine Carboxypeptidase DacA and DacB

Genomic DNA of the strain ANSB168 was isolated using a Bacterial Genomic DNA Extraction Kit (Transgen, China). Then, it was used as a template for cloning and the d-alanyl-d-alanine carboxypeptidase DacA and DacB genes were amplified by PCR. A primer pair of 5′-GTAGATTCATATGGCCAGCGATCC-3′ (forward primer) and 5′-CATCTCGAGAAACCAGCCGGTTA-3′ (reverse primer) was used for DacA, while another primer pair of 5′-GCTTATTCATATGGCTATAGATGTC-3′ (forward primer) and 5′-CATCTCGAGTATTGACCATTTTG-3′ (reverse primer) was employed for DacB. With the same restriction enzyme sites of *NdeI* and *XhoI* (underlined), the PCR amplification of these two carboxypeptidases was carried out separately. The conditions of PCR were 3 min initial denaturing at 9 °C, followed by 35 cycles of 30 s denaturing at 95 °C, 30 s annealing, and 90 s extension at 72 °C, and then a final extension at 72 °C lasting for 15 min. A TIANgel Midi Purification Kit (Tiangen, Beijing, China) was used to recycle the PCR product. By using an Omega D6943-01 Plasmid Mini Kit I (Tiangen, Beijing, China), the pET-31b vector was digested by *NdeI* and *XhoI*. The PCR products were also digested by the same restriction sites, and then they were cloned into digested pET-31b vectors (T4 DNA Ligase, New England Biolabs, MA, USA). After being authenticated in the *E. coli* DH5α strain, the selected colonies were transformed into an *E. coli* Rosetta strain (DE3).

### 4.4. Heterologous Expression and Protein Purification

The manipulated cells with pET-31b-DacA/pET-31b-DacB vectors were cultured in LB broth (HuanKai, Guangdong, China) at 3 °C with an agitation of 180 rpm, and then IPTG was added to the broth with a cell at OD_600_ 0.6 to induce for 4 h. The fermentation broth was centrifuged at 12,000 rpm for 30 min and redissolved in binding buffer, followed by ultrasonication and 10 min of centrifugation, and then the supernate and sediment were collected separately. The overexpression of the target protein was measured by using (SDS-PAGE. Because the recombinant protein has a His-tag, a nickel-nitrilotriacetic acid-agarose resin (Ni-NTA, Qiagen, CA, USA) was applied based on affinity chromatography for purification. After loading the sample, the column was purified by a different portion of binding buffer (50 mM sodium phosphate, 500 mM NaCl, 50 mM imidazole, pH 7.4) and elution buffer (50 mM sodium phosphate, 500 mM NaCl, 300 mM imidazole, pH 7.4). The purified protein was analyzed by SDS-PAGE as well as Western blotting and concentrated by ultrafiltration.

### 4.5. SDS-PAGE and Western Blot Analysis

SDS-PAGE and Western blot analysis were conducted by previously reported methods [55]. The first antibody was a mouse anti-His antibody, and the second antibody was goat anti-mouse IgG (H+L)–horseradish peroxidase (HRP, 1:2000, Abcam, Cambridge, UK).

### 4.6. OTA Degradation Activity and Relevant Enzyme Characteristics

OTA working solution was prepared using pure methanol and the final concentration was 2 μg/mL. The recombinant proteins with the final concentration of 400 μg/mL were added into different degradation systems. The reaction system had a 500 μL volume consisting of 10 μL of OTA standard (100 μg/mL in methanol) and 490 μL of reagents. The reaction system without recombinant DacA and DacB was regarded as a negative control. To determine the optimal pH, varying pH values (5.5, 6.0, 6.5, 7.0, 7.5, 8.0, 8.5) were selected in 0.1 M buffer (citric acid-sodium citrate buffer, pH: 5.5–6.5; disodium hydrogen phosphate-sodium dihydrogen phosphate buffer, pH: 6.5–8.5). At the optimal pH DacA or DacB, a 500 μL volume reaction system was applied to assess optimal temperature (27 °C, 32 °C, 37 °C, 42 °C, 47 °C). The degradation solution had a 500 μL volume and was incubated in total darkness for 72 h at 37 °C; then, 500 μL of HPLC grade methanol was used to terminate the reaction. The OTA degradation dynamic was explored at optimal pH and temperature and incubating for 5 days. The OTA degradation ability and degraded product were analyzed using HPLC. 

### 4.7. Dietary Treatments of Animal Trial

The trial was conducted with a basal diet that was used as a negative control and also to produce the OTA contaminated dietary treatments. An *A. ochraceus* (CGMCC 3.4412) strain was used to produce OTA by artificial infection of sterile maize for 21 d at 25–28 °C, and then the maize was dried and smashed. The concentration of OTA in maize powder was measured by HPLC, which was later added into the basal diet at 18% to meet the predicted concentration and verified by HPLC (predicted: 250 μg/kg, measured: 247.8 μg/kg). The broth of strain ANSB168 was transformed into a freeze-dried powder. The number of bacteria in the powder was later determined as 3 × 10^7^ CFU/g. Then, it was added to the contaminated basal diet up to an overdose of 2 kg/T feed to ensure the effectiveness of degradation.

### 4.8. Animal Trial in Layers

All procedures were reviewed and approved by the Laboratory Animal Welfare and Animal Experimental Ethical Committee of China Agricultural University (No. AW 13301202-1-7). The trial strictly complied with the standard operating procedures for experimental animals of the Ministry of Science and Technology (Beijing, China), and every effort was made to minimize suffering.

A total of 45 Jingfen No. 1 layers (26 weeks of age) were randomly allocated to three feeding treatments and divided between 15 pens. The nutritive values and feeding procedures referred to the NY/T 33-2004 (China) and recommendations for Jingfen No. 1 layers (Huadu yukou, Beijing, China). For the first 7 days, all birds were fed a basal diet. From day 8 onwards, the birds on the control diet continued a basal diet while the other treatment groups received a targeted 250 μg/kg OTA-contaminated diet. An amount of 2 kg/T freeze-dried bacterial powder (ANSB168) was added to one of the OTA-exposure groups. The production performance of laying hens was continuously calculated over a period of 1–28 days.

### 4.9. Analysis of Serum Parameters

The contents of AST, ALT, ALP, PEPCK, Cr, TP, ALB, LZM, T-AOC, SOD, and GR in serum were measured using diagnostic kits (Nanjing Jiancheng Bioengineering Institute, Nanjing, China) according to the manufacturer’s instructions. The activity of LAP and AAP, as well as the levels of MDA, T-GSH, GSH-Px, globulin (β2-MG, IgA, IgG, IgM), and cytokines (TNF-α, IL-2 and IL-10) in the serum were measured with the ELISA kit (Nanjing Jiancheng Bioengineering Institute, Nanjing, China). All procedures were performed according to the manufacturer’s instructions.

### 4.10. Extration and Clean-Up of OTA in Feed and Eggs

Levels of OTA in feed were determined before the animal trial. Residues of OTA and OTα in eggs were determined weekly during the animal trial, i. e., days 14, 21, and 28. OTA extraction from feed and eggs was conducted according to the methods of previous studies [49,56]. Then, extracted samples were passed through immunoaffinity clean-up columns (OchraTestWB; VICAM, Watertown, MA, USA) at a rate of 1–2 drops s^−1^ under gentle pressure provided by a vacuum clean-up assembly. The column was washed with 10 mL of water–methanol (90:10, *v*/*v*) and then dried under nitrogen gas (N_2_) for 5 min. Finally, OTA was eluted from the column by passing 2.0 mL of pure methanol [57].

### 4.11. High-Performance Liquid Chromatography

The contents of OTA and OTα were determined using an HPLC system (Shimadzu LC-10 AT, Shimadzu, Tokyo, Japan) equipped with an Agilent^®^ Eclipse Plus C18 column (5 μm, 4.6 × 150 nm) and a fluorescence detector (Shimadzu RF-20A, Shimadzu, Tokyo, Japan). Samples were filtered using a 0.22 μm filter and 20 μL of volume was loaded to the HPLC system. OTA and OTα detection used 333 nm and 477 nm as the wavelengths of excitation and emission, respectively. The mobile phase consisted of acetonitrile–water–glacial acetic acid (99:99:2, *v*/*v*/*v*), and the flow rate was 1 mL min^−1^ [57]. The limits of detection in feed and egg samples (defined as signal/noise ratio = 3) were estimated to be 0.1 μg/kg for both OTA and OTα.

### 4.12. Statistical Analysis

Statistically significant differences between mean values of the parameters tested in the animal trial were analyzed with ANOVA using Tukey’s honestly significant difference (HSD) post-hoc test in the SPSS statistical software package (version 22, Armonk, NY, USA). The differences were considered statistically significant if the *p* values < 0.05, although *p* values < 0.10 have also been used to demonstrate a trend because of the small number of subjects. The GraphPad Prism software for Windows was used to generate graphs (version 900, San Diego, CA, USA).

## 5. Conclusions

In summary, we elucidated the mechanism of an isolated OTA-degrading strain *Bacillus subtilis* ANSB168 by cloning and expressing the D-Ala-D-Ala carboxypeptidase DacA and DacB from ANSB168, which broke the amide bond of OTA to generate low-toxic metabolites OTα. In the animal trial, 250 μg/kg of OTA in the diet caused significant damage to the kidney and liver, induced oxidative stress, caused obvious inflammation, and activated the humoral immune system in the layers, which led to a decline in animal fitness. OTA degradation agent ANSB168 bioproducts were able to alleviate OTA-induced kidney and liver damage; relieve OTA-induced oxidative stress, immune response, and inflammation; and ultimately improve the health of animals. The detoxification mechanism of the strain ANSB168 involves mainly reducing the content of OTA by degrading mycotoxin in the intestine, thus alleviating its negative effects. The optimization of the ANSB168 bioproducts needs to be further developed in the future.

## Data Availability

Publicly available datasets were analyzed in this study. This data can be found here: https://www.ncbi.nlm.nih.gov/nuccore/OK663194.1/, accessed on 4 November 2021.

## Abbreviations

OTA: ochratoxin A; OTα: ochratoxin α; Phe: L-β-phenylalanine; CPA: carboxypeptidase A; CPB: carboxypeptidase B; HPLC: high-performance liquid chromatography; NCBI: National Center for Biotechnology Information; IPTG: isopropyl-b-D-thiogalactoside; SDS-PAGE: sodium dodecyl sulfate-polyacrylamide gel electrophoresis; LAP: leucine aminopeptidase; AAP: alanine aminopeptidase; PEPCK: phosphoenolpyruvate carboxykinase; Cr: creatinine; AST: aspartate aminotransferase; ALT: alanine aminotransferase; ALP: alkaline phosphatase; MDA: malonaldehyde; T-GSH: total glutathione; GSH-Px, glutathione peroxidase; T-AOC: total antioxidant capacity; SOD: superoxide dismutase; TAS: total antioxidant status; GR: glutathione reductase; TP: total protein; ALB: albumin; β2-MG: β2-microglobulin; IgA, IgG, IgM: immunoglobulin A, G, M; LZM: lysozyme; IL-2: cytokines like interleukin-2; IL-10: interleukin-10; TNF-α: tumor necrosis factors-α.
